# Socioeconomic status, well-being and mortality: a comprehensive life course analysis of panel data, Germany, 1984-2016

**DOI:** 10.1186/s13690-021-00559-7

**Published:** 2021-03-24

**Authors:** Diego Montano

**Affiliations:** Institute of the History, Philosophy and Ethics of Medicine, Department of Medical Sociology, Ulm University, Parkstr. 11, Ulm, 89073 Germany

**Keywords:** Longevity, Socioeconomic determinants of health, Life satisfaction, Satisfaction with health, Longitudinal study

## Abstract

**Background:**

This study seeks to explore potential causal mechanisms involved in the observed associations between several socioeconomic status (SES) indicators, well-being and mortality, by taking a life course perspective focusing on (i) the trajectory of income and domain-specific well-being indicators, (ii) the influence of different SES indicators on well-being and mortality, (iii) the interactions between those trajectories, and (iv) the associations of the income and domain-specific well-being trajectories with all-cause mortality.

**Methods:**

Socioeconomic status is operationalised by net household income, education, employment and marital status. Well-being is measured with two indicators: life satisfaction and satisfaction with health. Data from the German Socio-Economic Panel, collected between 1984 and 2016 and comprising more than 55,000 individuals, are analysed by means of longitudinal *k*-means cluster analysis, simultaneous equation systems and parametric time-to-death regressions.

**Results:**

The analyses indicate the presence of large reciprocal effects of the trajectories of income and well-being on each other. However, the results suggest that well-being has a larger influence on income than the opposite, namely, income on well-being. The mortality analysis, on the other hand, revealed that the history of satisfaction with health is a much stronger predictor of longevity than the individual’s income history. Mortality risk was found lower among married individuals and those with tertiary education. In contrast, unemployment was associated with lower income and well-being levels. The findings provide support to the notion that education is a superior SES indicator than income in the investigation of the social determinants of well-being and mortality.

**Conclusion:**

The present study provides evidence of large reciprocal effects of income and well-being and emphasises the importance of taking a life course approach in the investigation of the social determinants of health. Several SES indicators and both well-being indicators were found to be highly predictive of all-cause mortality and indicate the presence of cumulative effects related to one’s income and well-being trajectories.

## Background

The explanation of the associations between socioeconomic status (SES) and health usually centres around three major causal hypotheses: (i) SES is a cause of health outcomes, (ii) health outcomes are causes of SES, or (iii) SES and health outcomes are jointly caused by a third factor [54]. Previous research has found support for both causal directions, namely, from SES to health [54], and vice-versa, from health to SES [1, 44]. Other studies have focused on genetic predisposition as a third factor explaining the correlation of both SES and health in twins samples. These studies have resulted likewise in mixed evidence showing either reduced genetic variance with income levels, i.e., SES has an additional causal influence on health [31], better health outcomes among twins in higher SES levels in comparison to their twin siblings of lower SES [35], positive effects of social capital on health after adjustment of genetic factors [16], but also small or no effects of SES on different health outcomes [17, 47].

On the other hand, several studies have provided evidence of a large association between measures of self-rated health and well-being with mortality, whereby the death risks decrease with increasing levels of self-rated health and well-being [27, 28, 40, 51]. This observation is important in the context of the social determinants of health, since it evidences the importance of the cognitive appraisals of one’s own health as a predictor of objective health outcomes and, therefore, alludes to potential mechanisms by means of which social constructs such as SES may exert an influence on specific health outcomes. As cognitive appraisal processes of past and current health states and life events, well-being and self-rated health are intricately related to the social environment of individuals [10, 11, 32], and, therefore, there should be some causal links relating SES, well-being and health. Moreover, the concept of well-being itself can be further subdivided in domains specifically related to appraisals of either mental or physical health [10]. In this regard, the associations of well-being and mortality may also depend on how the wording of particular well-being indicators elicits domain-specific appraisals. For instance, previous research has suggested that self-rated health items such as “how would you say your health is?” are more related to the mental health domain than the physical health domain [2]. Nevertheless, to the knowledge of the author, the investigation of how different, domain-specific well-being indicators are related to mortality and SES has been rather limited so far [11, 14].

Despite the great methodological difficulties posed by the complex causal structure determining the relationship between SES, domain-specific well-being indicators and mortality, one feasible approach to gain some insight into this complexity consists of taking a multidimensional, life course perspective and, thereby, explicitly considering the path dependence of SES and well-being indicators and their reciprocal influence on mortality. To some extent, the life course approach is similar to the assumption that the present values of some quantity *X*_*t*_ at time *t* depend from the values taken by the same quantity in the previous *k* time points, say *X*_*t*−*k*_, i.e., the so-called *k*-order Markov process [1, 44]. By considering the individual’s history of SES and well-being indicators, it is possible to explore the interactions between several SES indicators, domain-specific well-being and mortality from the perspective of the temporal path dependence of the variables involved. A life course approach allows the estimation of cumulative or persistent tendencies throughout the life span and facilitates a more efficient statistical analysis of potential pathways linking predictors and outcomes [3, 43].

Even though most of the aforementioned literature has been dedicated exclusively to the relationship between income and health, the SES construct is actually a multidimensional one involving a series of complex causation pathways [34]. The present study not only builds on previous research investigating the relationship between income and health outcomes, but also aims to expand the scope of analysis by considering the interaction effects of income, domain-specific well-being indicators and the multidimensional character of the SES construct. In particular, this study seeks to explore potential causal mechanisms involved in the observed associations between SES indicators, well-being and mortality, by taking a life course perspective focusing on (i) the trajectory of income and domain-specific well-being indicators, (ii) the influence of different SES indicators on well-being and mortality, (iii) the interactions between those trajectories, and (iv) the associations of the income and domain-specific well-being trajectories with all-cause mortality.

### Research hypotheses

It has been observed that the association between income and well-being depends both on the individual’s own absolute income level, and the subjective perception of how one’s income relates to the incomes of others [12, 50]. From a life cycle perspective it has been argued that income growth does not cause a proportional increase in well-being, because material aspirations could also rise in proportion to income, thereby offsetting to some extent the income effects on well-being (plateauing effect) [13]. Moreover, some evidence suggests that the effects of absolute income on health may be more important for lower incomes than for higher ones [5]. Hence, it is hypothesised that:

**Hypothesis 1**: The income effects on well-being are greater among individuals in low income trajectories than among those in higher trajectories.

Furthermore, given that the appraisal of psychological well-being has also been shown to be positively related to income [33], there might be interaction effects between both well-being indicators, so that:

**Hypothesis 2**: The income effects on satisfaction with life are less among individuals who are more satisfied with their health than those who are not.

On the other hand, there is evidence showing that health symptoms, illnesses and/or disability may conduce to substantial income losses, reduction of work hours, unemployment and poverty risk [6, 8, 46, 60]. Since increasing income does not seem to imply unlimited increasing levels of well-being as stated above, it can expected that:

**Hypothesis 3**: The effects of the well-being indicators on income are greater than the effects of income on well-being.

Furthermore, since the likelihood of illnesses and all-cause mortality may be associated with particular behaviours and cognitions developed in the (social) context of the educational system, it can expected that the educational level has a larger explanatory power regarding all-cause mortality than income does. Results from large cross-country analyses seem to confirm that education has a stronger explanatory power than income regarding the rates of (preventable) chronic diseases and mortality [42, 58]. Hence, it is expected that:

**Hypothesis 4**: The effects of education on well-being and mortality are greater than the corresponding income effects.

It has been acknowledged that social relationships may exert an influence over longevity through various factors including social roles, social support, the quality of available social relationships and income itself [26]. Besides being an indicator of the social standing of an individual regarding the social norms of close, family-related relationships [38, 59], marital status is also an indicator of close affective relationships as well, for which a positive association with longevity has been reported, albeit not so among conflict-ridden marriages [37]. Furthermore, the mechanisms whereby unemployment may negatively affect health are multiple and include aggravation of social isolation, low income, limited access to health-related services or poor housing, which ultimately would lead to poor health outcomes [7, 45, 48]. Hence, it is hypothesised that:

**Hypothesis 5**: (a) Married individuals live longer and report higher levels of well-being than singles and non-married individuals, except for the separated and divorced, and (b) unemployed individuals report lower levels of well-being.

Finally, as stated in the Background section, the fact that subjective measures of health such as well-being have been found to be strong predictors of mortality [22, 49, 55] underlines the diagnostic relevance of the cognitive appraisal of one’s own health functioning. Given that those appraisals convey information on perceived vitality and the extent of health functioning decline, it can hypothesised that:

**Hypothesis 6**: The effects of well-being on all-cause mortality are greater than the corresponding income effects.

## Methods

### Data

The research hypotheses are investigated with data from the German Socio-Economic Panel Survey (SOEP) collected between 1984 and 2016. SOEP is an ongoing representative panel survey of private households in the Federal Republic of Germany which includes several socioeconomic variables at the household and individual level [23]. SOEP’s samples are typically obtained in a two-stage stratified sampling from the target population. In the present investigation data from individuals aged 14 to 100 years are analysed.

### Variables

*Net household income* is collected as monthly net household income in Euro. In order to compare income levels across waves, the net household income was adjusted for inflation with the German consumer price index (basis year 2011). *Satisfaction with life and health* are measured by two 10-category Likert items: “How satisfied are you at the present with your life, all things considered?” and “How satisfied are you with your own health?”, respectively (from 0: completely unsatisfied, to 10: completely satisfied). Well-being is understood as the subjective experience of happiness, contentment or desired mental states [57], and is measured by two indicators, namely, satisfaction with life and satisfaction with health. These measures are *stricto sensu* attitudes, i.e., cognitive appraisals on the individual’s own life and health stemming from recollections of past events, affect, proprioception, perception and/or cognitions [15]. Although the SOEP datasets provide mental and physical component scores equivalent to the generic Short Form Health Survey (SF12v2), one of most common measures of health status, these are available every two years from 2002 until 2016 only. On the contrary, the items on satisfaction with life and health have been asked every year since the beginning of the survey in 1984. Whereas satisfaction with life correlates with the mental component scores (*r*=0.47), satisfaction with health is much more related to the physical component scores (*r*=0.66). Hence, both satisfaction measures can be looked upon not only as well-being indicators, but also as generic measures of perceived mental and physical health status [10, 39].

*Education* entered the analysis in the CASMIN classification comprising five educational levels: inadequately completed basic education, basic education, intermediate, maturity, and tertiary education [4]. *Marital status* included the four categories married, single, separated or divorced, and other. *Employment status* consisted of full-time employment, part-time, vocational training, irregular employment, and not employed. The socio-economic status is thus operationalised by net household income, educational level, and employment and marital status. *The year of death* is included for all persons who have been confirmed as deceased over the course of the SOEP surveys, irrespective of the cause of death. This variable is right-censored, i.e., it includes individuals still alive up to the survey for which data are available, persons whose exact whereabouts are unknown or dropped out of SOEP, or deceased persons whose year of death is unknown [52].

### Statistical analysis

The clustering of the trajectories of net household income and life and health satisfaction was performed by means of longitudinal *k*-means cluster analysis [20]. The main advantages of using this type of partitioning algorithm instead of model-based approaches such as latent-class analysis are their independence from parametric assumptions or shapes concerning the trajectories and a more robust numerical convergence [19]. In comparison to some cross-sectional approaches such as income quintiles, a longitudinal clustering provides information on the relative continuity of income differentials and, therefore, simultaneously conveys information on cross-sectional and longitudinal income differentials, i.e., who earns more or less at a given time point and who has continuously earning relatively more or less during the observation period, respectively. The logarithm of net household income was taken in order to counter the influence of very high incomes in the subsequent statistical analyses. Missing values were dealt with the adjustment proposed by Gower in which the Euclidean distance is weighted by the number of available observations. Individuals with at least two observations in each main outcome were considered during clustering. The choice of the number of clusters was established by considering the maximum value of the statistics of Calinski und Harabatz, Davies and Bouldin, and the Akaike’s Information Criterion (AIC) [20], and the parsimony of the partition regarding the number of all possible category combinations. Cluster analysis of longitudinal data consists of the estimation of the distance between individual trajectories, and the identification of the partition which minimises the within-variance $\sigma _{W}^{2}$ and, at the same time, maximises the between-variance $\sigma _{B}^{2}$ of distances between observations in the different clusters. Let *y*_*it*_=(*y*_*i*1_,…,*y*_*it*_) be the row vector of outcome *y*, i.e., net household income and life and health satisfaction, for individual *i* at SOEP survey *t*. The Euclidean distance *d*(*i,j*) of the trajectories of two individuals *i,j* can be thus defined as:

1 $$\begin{array}{*{20}l} d(i, j) & = \left(\frac{1}{t} \sum_{k = 1}^{t} (y_{{ik}} - y_{{jk}})^{2} \right)^{1/2}  \end{array} $$

2 $$\begin{array}{*{20}l} C(g) & = \frac{\sigma_{B}^{2}}{\sigma_{W}^{2}} \cdot \frac{n - g}{g - 1} \end{array} $$

where *g* is the number of clusters for which the partition *C*(*g*) of the sample *n* is maximised [20].

Since the investigation of the research hypotheses 1, 2, 3 and 5 involves the simultaneous consideration of different SES indicators (i.e., a system of simultaneous regression equations), the so-called seemingly unrelated regressions (SUR) model offers the appropriate framework to estimate efficiently the corresponding regression coefficients [61]. The basic assumption of this type of models is that the residual variances of each regression equation in the equation system are correlated. The estimation of variance is performed by the method of generalised least squares which takes into account the covariance structure of the equation system and, at the same time, leads to more efficient estimates of the regression coefficients than an ordinary least squares regression performed on each equation separately [53]. The equation systems in the present investigation were estimated with the last complete observation of the survey participants (*N*=57,112) and are specified for three dependent variables, namely, the logarithm of net household income, and satisfaction with life and health, respectively.

For each dependent variable two equation systems M1 and M2 are calculated separately and adjusted for year of data collection (see below). Model (M1) includes main effects of the trajectory clusters, and model M2 extends M1 by including cluster interactions. In models M1 and M2, all clusters entered the regressions with the exception of the clusters of the same outcome variable in order to investigate the research hypotheses regarding the relative contribution of the trajectories to the explained variance of the other magnitudes. For instance, if household income (IN) is the dependent variable, only the clusters (CL) of satisfaction with life (SL) and satisfaction with health (SH) are considered in the interaction models. A similar procedure was applied for the regression equations of the well-being indicators. In order to ease the interpretation of results, the dependent variables were standardised before estimating the equation system. The following SUR models were estimated:

$$\begin{array}{*{20}l} & \text{SUR Model M1} \left\{\begin{array}{ll} IN = \alpha_{1} X^{T} + \alpha_{2} CL_{{SL}} + \alpha_{3} CL_{{SH}}  \\ SL = \alpha_{1} X^{T} + \alpha_{2} CL_{{SH}} + \alpha_{3} CL_{{IN}}  \\ SH = \alpha_{1} X^{T} + \alpha_{2} CL_{{SL}} + \alpha_{3} CL_{{IN}}  \end{array}\right. \\ & \text{SUR Model M2} \left\{\begin{array}{ll} IN = \alpha_{1} X^{T} + \alpha_{2}(CL_{{SL}} \times CL_{{SH}})  \\ SL = \alpha_{1} X^{T} + \alpha_{2} (CL_{{IN}} \times CL_{{SH}})  \\ SH = \alpha_{1} X^{T} + \alpha_{2} (CL_{{IN}} \times CL_{{SL}})  \end{array}\right. \end{array} $$

with *X*^*T*^ a predictor matrix with age and gender, and additionally, for the fully adjusted models, the SES indicators education, employment status and marital status. The vector of regression coefficients *α*=(*α*_1_,...,*α*_5_)^*T*^ of the SUR models is estimated by the method of generalised least squares as [61]:

$$\begin{array}{*{20}l} \alpha = \left(X^{T} \Sigma^{-1} X \right)^{-1} X^{T} \Sigma^{-1} Y, \quad Var(\alpha) = \left(X^{T} \Sigma^{-1} X \right)^{-1}, \end{array} $$

with *Σ* being the covariance matrix, *X* the matrix of predictors and *Y* the stacked vector of dependent variables, namely, income, and life and health satisfaction. In the estimation of the SUR models it is assumed that the error terms *σ*_*i*_,*σ*_*j*_,*i*=1,2,3, of the regression equations are correlated, yielding *Σ*=*Σ*_*d*_⊗*I*_*T*_, with the disturbance covariance matrix *Σ*_*d*_=*σ*_*i*_*σ*_*j*_, the Kronecker product ⊗, the identity matrix *I*_*T*_, and the number of observations *T* in each equation [25].

The mortality analysis was based on parametric time-to-death regressions using the Gompertz hazard function [24]. This parametrisation belongs to the so-called accelerated failure time models in which it is assumed that ageing gradually increases the probability of death between survey waves (i.e., censoring time) according to the specific effects of the set of covariates. The Gompertz hazard function, *h*(*t*|*λ*,*γ*(*X*)), parametrises the mortality rate as:

3 $$\begin{array}{*{20}l} h(t|\lambda, \gamma (X)) & = \lambda e^{\gamma (X) t}, \quad \text{with} \end{array} $$

4 $$\begin{array}{*{20}l} \log [\gamma (X)] & = \beta X^{T}  \end{array} $$

with shape and rate parameters *λ*,*γ*, covariates *X* and regression coefficients *β* [30].

For the mortality analysis two models are estimated: In model S1 the trajectory clusters of income, satisfaction with life, satisfaction with health, age and gender are included as covariates. In model S2, marital status and education are added to the regression equations in order to test the corresponding research hypotheses. The regression coefficients of the parametric time-to-death analysis are reported in exponential form *e*^*β*^, where values greater than 1 indicate a higher probability of dying (“accelerated time to death”). The reported confidence intervals in the SUR models and the time-to-death regressions were estimated at the 99% level with the aim to reduce the probability of false positives for small effects [29]. *P*-values are not supplied since they give poor information about the likely result of a future replication [9]. All data handling and statistical analyses were performed with the statistical environment R (v. 3.6.2), especially the packages kml, systemfit and flexsurv [20, 25, 30].

## Results

**Fig. 1:**
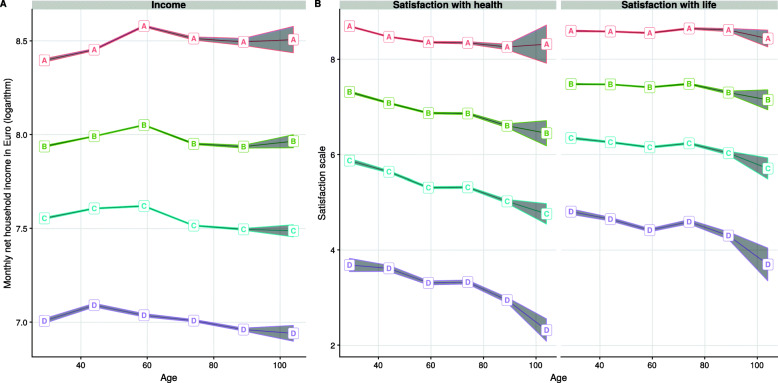
Average outcome values within trajectory clusters by 15-years age intervals and 99% confidence stripes. Panel A: net household income. Panel B: well-being indicators. German SOEP 1984-2016

According to the Calinski und Harabatz, Davies and Bouldin, and AIC criteria, the optimal number of clusters for the trajectory partitioning of household income, and satisfaction with life and health varied between three and four clusters. However, in order to allow a more detailed analysis of the various trajectory patterns, the four-cluster solution was chosen for all three main outcomes. The sample proportions of each cluster and the means of the corresponding variables in the clusters are reported in Table 1 and Fig. 1. The average trajectory length per individual over all clusters was about $\bar {t} = 9.11$ years. Although the variance within clusters increases due to sample attrition (i.e., wider confidence stripes with increasing age in Fig. 1), the differences and trends between trajectories are quite stable during the life-span for both household income and well-being clusters.

**Table 1:** Descriptive statistics of the dataset in long format

Variable	Statistic	Missing values
**Gender**		0
Males	47.46 (263193)	
Females	52.54 (291391)	
**Education**		11844
Inadequately completed	4.04 (21938)	
Basic	40.49 (219739)	
Intermediate	25.80 (140019)	
Maturity	11.34 (61551)	
Tertiary	18.33 (99493)	
**Employment status**		11
Full-time	42.38 (235030)	
Part-time	10.72 (59477)	
Vocational	2.83 (15682)	
Irregular	4.03 (22359)	
Not employed	40.04 (222025)	
**Marital status**		3212
Married	62.71 (345742)	
Single	21.78 (120112)	
Sep./Divorced	9.16 (50494)	
Other	6.35 (35024)	
**Income clusters**		0
A - Highest income ($\bar {t}$ = 8.4 years)	15.43 (85545); *μ*= 8.50 (0.38)	
B - Higher income ($\bar {t}$ = 9.6 years)	36.94 (204852); *μ*= 7.99 (0.30)	
C - Lower income ($\bar {t}$ = 9.4 years)	34.50 (191341); *μ*= 7.57 (0.33)	
D - Lowest income ($\bar {t}$ = 8.2 years)	13.14 (72846); *μ*= 7.02 (0.43)	
**Satisfaction with life clusters**		0
A - Highest satisfaction ($\bar {t}$ = 7.5 years)	22.44 (124435); *μ*= 8.59 (1.01)	
B - Higher satisfaction ($\bar {t}$ = 9.6 years)	39.31 (217980); *μ*= 7.45 (1.20)	
C - Lower satisfaction ($\bar {t}$ = 10.4 years)	27.87 (154540); *μ*= 6.22 (1.54)	
D - Lowest satisfaction ($\bar {t}$ = 9.0 years)	10.39 (57629); *μ*= 4.56 (1.89)	
**Satisfaction with health clusters**		0
A - Highest satisfaction ($\bar {t}$ = 7.4 years)	28.47 (157871); *μ*= 8.50 (1.21)	
B - Higher satisfaction ($\bar {t}$ = 10.0 years)	36.23 (200936); *μ*= 7.00 (1.60)	
C - Lower satisfaction ($\bar {t}$ = 10.9 years)	26.17 (145134); *μ*= 5.41 (1.87)	
D - Lowest satisfaction ($\bar {t}$ = 9.0 years)	9.13 (50643); *μ*= 3.31 (2.09)	
Age	46.8 (17.0)	7
Net household income (logarithm)	7.8 (0.6)	7076
Satisfaction with life	7.1 (1.8)	4294
Satisfaction with health	6.7 (2.3)	3909
Observations	554,584	
Individuals	57,112	

Statistics: proportions (%) and frequencies in parentheses for categorical variables; mean (*μ*) and standard error in parentheses for metrical and ordinal variables. $\bar {t}$: average trajectory length per individual. German SOEP 1984-2016

**Fig. 2:**
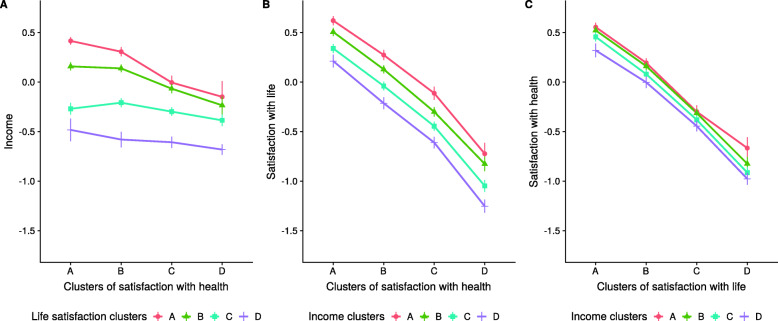
Fitted values of the seemingly unrelated regression (SUR) of model M2 in Table 3 and 99% confidence bars (interaction effects plots). For all cluster variables, namely, income, life satisfaction and satisfaction with health, the labels A to D represent an ordered scale going from A: highest level to D: lowest level. German SOEP 1984-2016

The estimated regression coefficients corresponding to the SUR models M1 and M2 are reported in Tables 2 and 3, respectively. In order to ease the interpretation of results from the interaction model M2, only coefficients excluding zero in the 99% confidence intervals are reported. In addition, Fig. 2 provides for the fully adjusted model M2 from Table 3 the fitted values of the main outcomes by trajectory clusters in a set of three interaction plots. For instance, panel A in Fig. 2 depicts on the x-axis the clusters of satisfaction with health (from A: highest satisfaction to D: lowest satisfaction) and on the y-axis the estimated logarithm of income, stratified by the clusters of satisfaction with health (four lines). Thus, panel A shows that for individuals in cluster D of the life satisfaction trajectories (bottom line), the income level is rather stable across the levels of satisfaction with health (x-axis, from A: highest satisfaction with health to D: lowest satisfaction with health), thereby indicating no interaction effect between life and health satisfaction.

**Table 2:** Seemingly unrelated regression (SUR) models on standardised dependent variables

	Income		Satisfaction with life		Satisfaction with health	
Variable	M1	M2	M1	M2	M1	M2
Intercept	-9.100 (-11.638; -6.563)	-8.332 (-10.874; -5.791)	-4.221 (-6.574; -1.868)	-4.342 (-6.696; -1.987)	-3.469 (-5.662; -1.276)	-3.417 (-5.618; -1.217)
Age	-0.007 (-0.007; -0.006)	-0.007 (-0.008; -0.006)	0.004 (0.003; 0.004)	0.004 (0.003; 0.004)	-0.018 (-0.019; -0.018)	-0.018 (-0.019; -0.018)
Females	-0.142 (-0.162; -0.122)	-0.142 (-0.162; -0.121)	0.075 (0.056; 0.094)	0.075 (0.056; 0.094)	-0.055 (-0.073; -0.037)	-0.055 (-0.073; -0.037)
**Main effects**						
IN-B (ref. A)			-0.152 (-0.180; -0.125)	-0.127 (-0.169; -0.086)	-0.069 (-0.096; -0.043)	-0.089 (-0.132; -0.046)
IN-C			-0.347 (-0.375; -0.318)	-0.312 (-0.357; -0.268)	-0.084 (-0.111; -0.057)	-0.087 (-0.134; -0.039)
IN-D			-0.547 (-0.581; -0.512)	-0.456 (-0.517; -0.394)	-0.102 (-0.135; -0.069)	-0.147 (-0.215; -0.078)
SH-B (ref. A)	0.017 (-0.009; 0.043)	-0.031 (-0.076; 0.014)	-0.401 (-0.424; -0.378)	-0.365 (-0.417; -0.313)		
SH-C	-0.053 (-0.087; -0.020)	-0.266 (-0.342; -0.190)	-0.829 (-0.857; -0.802)	-0.754 (-0.821; -0.687)		
SH-D	-0.051 (-0.098; -0.003)	-0.372 (-0.553; -0.192)	-1.437 (-1.475; -1.398)	-1.378 (-1.489; -1.268)		
SL-B (ref. A)	-0.260 (-0.286; -0.233)	-0.300 (-0.337; -0.263)			-0.362 (-0.385; -0.340)	-0.372 (-0.420; -0.323)
SL-C	-0.598 (-0.630; -0.567)	-0.754 (-0.814; -0.694)			-0.796 (-0.821; -0.770)	-0.879 (-0.945; -0.813)
SL-D	-0.929 (-0.973; -0.885)	-0.990 (-1.116; -0.864)			-1.294 (-1.328; -1.260)	-1.205 (-1.314; -1.096)
**Interaction effects**(only reported if 0∉ 99% CI)						
IN-B:SH-C				-0.088 (-0.168; -0.008)		
IN-B:SL-C						0.097 (0.018; 0.175)
IN-C:SH-C				-0.082 (-0.162; -0.003)		
IN-C:SL-D						-0.124 (-0.246; -0.002)
IN-D:SH-B				-0.108 (-0.195; -0.021)		
IN-D:SH-C				-0.145 (-0.241; -0.049)		
IN-D:SH-D				-0.169 (-0.303; -0.035)		
IN-D:SL-C						0.132 (0.034; 0.231)
SL-B:SH-B		0.082 (0.024; 0.141)				
SL-B:SH-C		0.198 (0.109; 0.287)				
SL-C:SH-B		0.153 (0.074; 0.233)				
SL-C:SH-C		0.384 (0.285; 0.482)				
SL-C:SH-D		0.480 (0.284; 0.676)				
SL-D:SH-C		0.258 (0.105; 0.412)				
SL-D:SH-D		0.378 (0.155; 0.601)				
*R*^2^	0.109	0.112	0.236	0.237	0.313	0.314
*ΔR*^2^(%)	2.240		0.176		0.060	

Regression coefficients and 99% confidence intervals in parentheses. Models: M1: main effects model, M2: interaction model. IN: net household income, SH: satisfaction with health, SL: satisfaction with life. N=57,112. *R*^2^ of the equation systems for the M1 and M2 models: 0.220 and 0.221, respectively. *ΔR*^2^: relative increase of R2 in percent between models M1 and M2 for the respective dependent variable. German SOEP 1984-2016

**Table 3:** Seemingly unrelated regression (SUR) models on standardised dependent variables and SES indicators

	Income		Satisfaction with life		Satisfaction with health	
Variable	M1	M2	M1	M2	M1	M2
Intercept	1.663 (-0.629; 3.954)	2.338 (0.042; 4.635)	-4.822 (-7.240; -2.405)	-5.586 (-8.008; -3.165)	-0.192 (-2.447; 2.062)	-0.432 (-2.704; 1.841)
Age	-0.003 (-0.003; -0.002)	-0.003 (-0.004; -0.002)	0.004 (0.003; 0.004)	0.004 (0.003; 0.004)	-0.016 (-0.017; -0.016)	-0.016 (-0.017; -0.016)
Females	0.016 (-0.004; 0.035)	0.012 (-0.008; 0.032)	0.091 (0.070; 0.112)	0.094 (0.073; 0.115)	-0.042 (-0.062; -0.022)	-0.042 (-0.061; -0.022)
**Education**						
Basic education (ref. no degree)	0.132 (0.084; 0.180)	0.129 (0.081; 0.177)	0.032 (-0.018; 0.083)	0.020 (-0.030; 0.071)	0.052 (0.004; 0.100)	0.053 (0.005; 0.101)
Intermediate	0.361 (0.311; 0.410)	0.358 (0.308; 0.407)	0.022 (-0.031; 0.074)	-0.027 (-0.079; 0.025)	0.098 (0.048; 0.147)	0.098 (0.048; 0.147)
Maturity	0.454 (0.401; 0.506)	0.446 (0.393; 0.499)	0.096 (0.040; 0.151)	0.035 (-0.021; 0.090)	0.107 (0.054; 0.159)	0.106 (0.053; 0.159)
Tertiary	0.784 (0.733; 0.835)	0.776 (0.725; 0.827)	0.132 (0.078; 0.186)	0.033 (-0.022; 0.087)	0.164 (0.113; 0.215)	0.159 (0.107; 0.210)
**Employment status**						
Part-time (ref. full-time)	-0.143 (-0.174; -0.112)	-0.142 (-0.173; -0.110)	-0.044 (-0.077; -0.011)	-0.030 (-0.063; 0.003)	-0.023 (-0.054; 0.008)	-0.023 (-0.054; 0.009)
Irregular	-0.419 (-0.463; -0.375)	-0.415 (-0.459; -0.371)	-0.106 (-0.153; -0.060)	-0.071 (-0.118; -0.025)	-0.012 (-0.056; 0.032)	-0.013 (-0.057; 0.032)
Vocational	-0.062 (-0.123; -0.002)	-0.066 (-0.126; -0.006)	0.080 (0.016; 0.143)	0.069 (0.006; 0.133)	-0.035 (-0.095; 0.025)	-0.037 (-0.097; 0.023)
Not employed	-0.532 (-0.556; -0.508)	-0.531 (-0.554; -0.507)	-0.108 (-0.133; -0.083)	-0.056 (-0.082; -0.031)	-0.137 (-0.161; -0.114)	-0.138 (-0.162; -0.114)
**Marital status**						
Single (ref. married)	-0.541 (-0.567; -0.515)	-0.531 (-0.557; -0.505)	-0.134 (-0.161; -0.106)	-0.068 (-0.096; -0.040)	0.045 (0.019; 0.071)	0.044 (0.017; 0.071)
Sep./Divorced	-0.743 (-0.773; -0.713)	-0.726 (-0.756; -0.696)	-0.272 (-0.304; -0.241)	-0.182 (-0.214; -0.150)	0.038 (0.008; 0.068)	0.038 (0.007; 0.069)
Other	-0.531 (-0.569; -0.493)	-0.525 (-0.563; -0.487)	-0.125 (-0.165; -0.084)	-0.048 (-0.089; -0.007)	0.044 (0.006; 0.082)	0.045 (0.006; 0.083)
**Main effects**						
IN-B (ref. A)			-0.123 (-0.151; -0.094)	-0.097 (-0.140; -0.055)	-0.032 (-0.060; -0.005)	-0.048 (-0.091; -0.005)
IN-C			-0.277 (-0.307; -0.247)	-0.241 (-0.287; -0.196)	-0.018 (-0.047; 0.010)	-0.015 (-0.063; 0.034)
IN-D			-0.419 (-0.457; -0.382)	-0.335 (-0.398; -0.272)	-0.022 (-0.059; 0.014)	-0.069 (-0.139; 0.001)
SH-B (ref. A)	0.007 (-0.017; 0.030)	-0.003 (-0.043; 0.036)	-0.399 (-0.422; -0.376)	-0.365 (-0.417; -0.313)		
SH-C	0.003 (-0.026; 0.033)	-0.120 (-0.186; -0.053)	-0.826 (-0.853; -0.798)	-0.751 (-0.818; -0.684)		
SH-D	0.084 (0.041; 0.126)	-0.150 (-0.309; 0.008)	-1.431 (-1.469; -1.392)	-1.359 (-1.470; -1.249)		
SL-B (ref. A)	-0.190 (-0.213; -0.167)	-0.215 (-0.248; -0.183)			-0.366 (-0.389; -0.344)	-0.370 (-0.419; -0.322)
SL-C	-0.429 (-0.457; -0.401)	-0.485 (-0.538; -0.432)			-0.796 (-0.821; -0.770)	-0.872 (-0.938; -0.805)
SL-D	-0.652 (-0.690; -0.613)	-0.606 (-0.716; -0.495)			-1.285 (-1.319; -1.251)	-1.192 (-1.301; -1.083)
**Interaction effects**(only reported if 0∉ 99% CI)						
IN-B:SH-C				-0.090 (-0.169; -0.010)		
IN-B:SL-C						0.087 (0.008; 0.165)
IN-C:SH-C				-0.083 (-0.163; -0.004)		
IN-C:SL-D						-0.132 (-0.254; -0.011)
IN-D:SH-B				-0.095 (-0.182; -0.008)		
IN-D:SH-C				-0.133 (-0.229; -0.037)		
IN-D:SH-D				-0.178 (-0.312; -0.045)		
IN-D:SL-C						0.134 (0.036; 0.232)
SL-B:SH-C		0.139 (0.061; 0.218)				
SL-C:SH-C		0.194 (0.107; 0.280)				
SL-C:SH-D		0.293 (0.121; 0.466)				
SL-D:SH-D		0.200 (0.003; 0.396)				
*R*^2^	0.316	0.317	0.242	0.243	0.318	0.319
*ΔR*^2^(%)	0.271		0.164		0.061	

Regression coefficients and 99% confidence intervals in parentheses. Models: M1: main effects model, M2: interaction model. IN: net household income, SH: satisfaction with health, SL: satisfaction with life. N=57,112. *R*^2^ of the equation systems for the M1 and M2 models: 0.291 and 0.293, respectively. *ΔR*^2^: relative increase of *R*^2^ in percent between models M1 and M2 for the respective dependent variable. German SOEP 1984-2016

The results of the SUR models in Tables 2 and 3 do not seem to provide sufficient support for Hypothesis 1, stating that the income effects on well-being are greater among individuals in low income trajectories than among those in higher trajectories. Albeit there are main income effects on life satisfaction, there were practically no income effects on satisfaction with health (Fig. 2, panels B and C; and Table 3, models of satisfaction with life and health, respectively). In addition, even though a few interactions of income and well-being were large (models M2), individuals in the lowest income level (cluster D) were not generally characterised by even lower levels of well-being, as expected if there were interaction effects detrimental to the lowest income clusters. Moreover, the estimates in Tables 2 and 3 indicate the presence of main effects of satisfaction with life on satisfaction with health, but only small interaction effects with the income clusters. These results are graphically illustrated in panel C of Fig. 2 in the overlapping marginal means of the corresponding curves.

In addition, since there are practically no interaction effects between income and satisfaction with health (parallel marginal means in Fig. 2, panel B), the levels of satisfaction with health do not moderate the association of income and satisfaction with life, thereby rejecting Hypothesis 2. On the other hand, the pairwise comparison of the the goodness-of-fit coefficients *R*^2^ between models M1 and M2 for each dependent variable in Tables 2 and 3 supports Hypothesis 3 in which it was expected that the effects of well-being on income are greater than those of income on well-being. This can be observed by considering that in all model specifications the relative increase of explained variance *ΔR*^2^ is greater when income is the dependent variable. Hence, the results indicate that income is more sensitive to the trajectory of the well-being indicators, especially life satisfaction, than the opposite, namely, the sensitivity of well-being to the income trajectories.

Furthermore, the results of the regression analyses provide some support for Hypothesis 4 stating that the effects of education on well-being and mortality are larger than the income effects: Individuals with higher educational levels report higher levels of satisfaction with life and health than individuals who inadequately completed basic education. In addition, higher educational levels were associated with lower all-cause mortality rates as suggested by the results of the parametric time-to-death analysis in Table 4, and contribute substantially to explained variance as suggested by the comparison of the AIC statistic of models S1 and S2.

**Table 4:** Parametric time-to-death analysis with Gompertz hazard function

	S1		S2	
Variable	Males	Females	Males	Females
**Main effects**				
IN-B (ref. A)	1.02 (0.84; 1.23)	0.71 (0.57; 0.89)	0.82 (0.68; 1.00)	0.63 (0.50; 0.80)
IN-C	1.11 (0.93; 1.33)	0.65 (0.53; 0.81)	0.79 (0.65; 0.97)	0.54 (0.43; 0.67)
IN-D	1.71 (1.41; 2.08)	1.12 (0.91; 1.38)	1.11 (0.89; 1.38)	0.85 (0.68; 1.07)
SH-B (ref. A)	1.78 (1.43; 2.22)	1.63 (1.24; 2.14)	1.73 (1.38; 2.17)	1.57 (1.19; 2.08)
SH-C	3.71 (2.96; 4.65)	3.40 (2.59; 4.45)	3.60 (2.86; 4.54)	3.27 (2.48; 4.32)
SH-D	8.56 (6.66; 10.98)	8.24 (6.17; 11.00)	7.90 (6.11; 10.22)	7.60 (5.63; 10.24)
SL-B (ref. A)	0.75 (0.64; 0.88)	0.70 (0.59; 0.84)	0.75 (0.64; 0.89)	0.71 (0.59; 0.85)
SL-C	0.73 (0.61; 0.87)	0.59 (0.49; 0.71)	0.75 (0.62; 0.90)	0.60 (0.49; 0.73)
SL-D	1.00 (0.81; 1.23)	0.81 (0.65; 1.00)	0.99 (0.80; 1.24)	0.82 (0.66; 1.02)
Age	1.08 (1.08; 1.09)	1.10 (1.09; 1.10)	1.09 (1.08; 1.09)	1.09 (1.09; 1.10)
**Education**				
Basic education (ref. no degree)			0.91 (0.69; 1.20)	1.18 (0.87; 1.60)
Intermediate			0.68 (0.50; 0.92)	0.95 (0.68; 1.32)
Maturity			0.68 (0.48; 0.97)	0.67 (0.44; 1.02)
Tertiary			0.42 (0.31; 0.57)	0.50 (0.34; 0.73)
**Marital status**				
Single (ref. married)			1.59 (1.27; 1.98)	1.64 (1.29; 2.10)
Sep./Divorced			1.16 (0.96; 1.41)	1.11 (0.89; 1.38)
Other			1.12 (0.96; 1.31)	1.20 (1.04; 1.39)
Person-years at risk	5,172,250	5,857,910	5,027,668	5,678,714
Observations	263,103	291,215	256,619	282,702
Events	2655	2266	2530	2156
AIC	37791	32020	35761	30352

Regression coefficients and 99% confidence intervals in parentheses. S1: reference model, S2: fully-adjusted model. IN: net household income, SH: satisfaction with health, SL: satisfaction with life. German SOEP 1984-2016

Concerning Hypothesis 5a stating that married individuals live longer and report higher levels of satisfaction with life and health than singles and non-married individuals, the estimates in Table 3 indicate that married individuals are more satisfied with life than individuals of other marital status categories. Nonetheless, in contradiction to Hypothesis 5a, married individuals report lower levels of satisfaction with health in comparison to individuals of the other marital status categories. At the same time, the estimates of the mortality analysis in Table 4 provide support to the notion that married individuals have increased longevity in comparison to singles, but not regarding the separated and divorced, or individuals reporting other marital status. Hence, Hypothesis 5a is only partially supported. In contrast, the results confirm the expected associations in Hypothesis 5b by showing that unemployed individuals report lower satisfaction with life and health than the full-time employed.

Finally, the results in Table 4 confirm Hypothesis 6 stating that well-being is a stronger predictor of longevity than income. This holds especially regarding the role of satisfaction with health as an indicator of well-being. In fact, the regression coefficients corresponding to satisfaction with health are largest. In model S1, males in the lowest income trajectories (cluster D) show a larger mortality risk than males in cluster A. Once education and marital status are considered, however, the mortality risks of males in the income clusters B and C decrease (especially in cluster C), and the differences between clusters A and D are not longer observed, thereby indicating mediation effects of both education and marital status on the association between income and mortality. Among women, the income effects in both models S1 and S2 are more stable and point consistently to increased mortality of females in clusters A and D, in comparison to clusters B and C.

## Discussion

In the present study six research hypotheses were investigated concerning the interactions of household income trajectories, education, and employment and marital status (as SES indicators) and satisfaction with life and health (as indicators of well-being), and their influence on present income levels, well-being and all-cause mortality. Hypotheses 1 and 2, in which a substantial contribution of income to well-being was assumed, were not confirmed; on the contrary, the results indicate that well-being, especially the life satisfaction domain, has a larger influence on income than income on well-being (Hypothesis 3). Given that the present study considers explicitly the history of income and well-being levels and, therefore, accounts to some extent for unobserved heterogeneity in the main outcomes, there is evidence that the variation of well-being levels is less sensitive to the history of the relative income position of individuals than the opposite (see *ΔR*^2^ values in Tables 2 and 3). Despite the fact that the interaction models M2 did not suggest, in general, the presence of large interaction effects between the income and well-being trajectories, there were substantial reciprocal main effects of the particular trajectories on each other. Furthermore, regarding overall mortality, the preponderance of well-being over income is underlined by the results of the time-to-death analysis in which longevity shows the strongest associations with perceived well-being (Hypothesis 6). For instance, the risk difference between the health satisfaction clusters A and D in models S2 would correspond on average to the ageing effects of roughly 24 years for both males and females, i.e., log(*β*_*SH*_)/ log(*β*_*age*_). Furthermore, by comparing the pattern of associations obtained in the SUR and mortality models, it becomes evident that the associations of SES and health are rather domain specific: Whereas life satisfaction has a stronger association with income than health satisfaction (Table 3), health satisfaction, in contrast, is a much better predictor of mortality than life satisfaction (Table 4). Since life and health satisfaction are related to some extent to the mental and physical health domains, respectively, the results suggest that the investigation of the relationships between SES and health would need the explicit consideration of the peculiarities of the different domain-specific health indicators, as already observed in previous research [49].

The results reported in Tables 3 and 4 provide support to the notion that education is a superior SES indicator in the investigation of the socioeconomic determinants of well-being and mortality (Hypothesis 4), as reported elsewhere [42, 58]. According to the findings of the present study, the estimated differences in mortality between the lowest and tertiary educational level would correspond to ageing effects of about 10 and 8 years for males and females, respectively, i.e., log(*β*_*educ*_)/ log(*β*_*age*_). Under consideration that the SES indicators marital status, employment and education not only capture to some extent aspects related to the individual’s meaning of life, but also account for a large proportion of explained variance of well-being and/or all-cause mortality, the argument that the associations of SES and health are multi-causal receives additional support. There does not seem to be a single SES factor accountable for variation in health outcomes; on the contrary, SES and health exert reciprocal influence during the life-span, and co-determine various aspects of the individual’s life history at the level of income, education, employment, social relationships, and well-being appraisals. It is noteworthy that the results of the present study resemble previous findings with data from the Stockholm Birth Cohort study, in which the constant co-occurrence of disadvantages during the life course in terms of social assistance (i.e., income losses), unemployment and mental illnesses was a stronger predictor of mortality than the occurrence of single disadvantages [56].

From a life course perspective on mortality, the present results provide evidence of the so-called terminal decline, i.e., the steep decline of health functioning shortly before death [21]. The pronounced decline in the survival probability before death occurs much earlier in the life course for individuals with a history of low levels of satisfaction with health (cluster D). At the same time, since adverse circumstances during childhood may result in reduced well-being levels in adult life, the positive effects of education and marital status on well-being and mortality (Table 3, models S2) seem to indicate that the socioeconomic achievements in adulthood may counterbalance to some extent adverse childhood experiences, as has been suggested previously [14].

Two results in the present study deserve further discussion: First, satisfaction with life does not show a dose-response relationship with mortality, but a non-linear U-shaped relationship with higher mortality risks for both clusters A and D among males and females. Second, in models S1 and S2, the all-cause mortality rates of females in the income clusters A (highest) and D (lowest) were higher in comparison to those in the middle-income clusters B and C (Table 4). Similarly, mortality rates of males in income cluster C were lower than for the reference cluster A, but only in the adjusted model S2. These results imply that the associations between specific SES indicators and health do not necessarily follow a gradient of better health for increasing SES levels for particular samples. In fact, analyses of mortality data at the population level indicate that the accumulation of particular health risks and illnesses may be greater among individuals of higher SES in some, but not all, countries [41].

The non-linear U-shaped relationship between satisfaction with life and mortality could be the result of increased, but qualitative different, mortality risks among individuals reporting either very high or very low levels of satisfaction with life (clusters A and D) in comparison to the middle ranges of the distribution (clusters B and C). Notice that previous findings concerning the associations of mortality and life satisfaction have been also mixed, suggesting either non-linear relationships or the limitations of the life satisfaction measurement in capturing the emotional dimension of the well-being construct [18]. The finding that for models S1 and S2 the mortality rates among females of income cluster A (highest) and cluster D (lowest) do not differ, may result from various reasons including a stronger sample attrition or increased longevity of females in the the middle-income trajectories (clusters B and C), underestimation of mortality for lower income females, or excess health risks operating at both extremes of the income distribution. Several explanations of the similar mortality rates between high and low income clusters may be related to certain behavioural patterns among high-income females which lead to excess death risk such as smoking in older cohorts, or more frequent exposure to traffic accidents or injuries, as suggested elsewhere [41].

### Limitations

The present study has two important limitations. First, life expectancy in the SOEP samples between 1995 and 2005 is higher than the corresponding estimates obtained from the official life tables for the whole German population, and amount to 85.4 vs. 75.3, and 84.4 vs. 81.3 years, for males and females, respectively [36]. Hence, the present findings may under- or overestimate the associations pertaining mortality, since death cases at earlier ages are underrepresented in SOEP. However, the conclusions obtained here are based on a relatively large sample size (more than 55,000 individuals), a long observation period (1984-2016) for the whole sample, and an average trajectory length of $\bar {t} = 9.11$ years per individual. Hence, the results are based on the most frequent values observable in the population in a relatively long period and, therefore, it is unlikely that a more comprehensive sampling of extreme values would completely invalidate the findings. On the contrary, the real SES and well-being differentials could be larger, e.g., if the very high mortality rates and very low incomes and well-being values among the homeless were to be considered. Nonetheless, it should be kept in mind that with the available SOEP sample strong effects of education, marital status, and well-being could be estimated at the very conservative 99% confidence level. Therefore, even if the real effects of income are larger than those reported in the present study, they should still be relatively small in comparison to the other SES indicators for which substantial differences were observed.

Second, due to the fact that there is substantial temporal autocorrelation of each variable with itself, the optimal cluster partitions according to the goodness-of-fit statistics tend to arrange the trajectories in ordered categories going from higher to lower levels of the main outcome (Fig. 1). Notice that the lack of clusters with crossing trajectories suggests a strong path dependence of the income and well-being levels. Forcing a cluster partition with more than four or five clusters in order to obtain clusters with crossing trajectories would violate the conditions of optimal cluster partitioning and, at the same time, increase the chances of non-reproducibility of results. Hence, it seems that strong and abrupt declines or increases of income and well-being levels are not characteristic at the population level, but would pertain rather to very specific circumstances among certain groups, or to consequences resulting from important adverse life events such as sudden job loss, severe injuries or chronic illnesses. Further analyses would be required to investigate the extent to which crossing trajectories (e.g., increasing vs. decreasing trajectories) in specific groups may be associated with changes in income, well-being levels and all-cause mortality rates.

## Conclusions

In the present investigation evidence was found that the history of well-being has a larger influence on the individual’s income history than the opposite. As hypothesised, education seems to be a better predictor than income regarding not only well-being levels, but also all-cause mortality. Similarly, in accordance with the hypothesis that social relationships are a pivotal resource in the life course, married individuals were associated with higher levels of income and satisfaction with health (but not with life), and increased longevity. Finally, the analyses revealed that the strongest predictors of all-cause mortality are the life history of satisfaction with life and health, the individual’s educational level, and the marital status. Given that these magnitudes are embedded in the socioeconomic circumstances during childhood development and young adulthood, the results are indicative of cumulative effects of several SES indicators and well-being on all-cause mortality.

## Data Availability

SOEP data is available from the SOEP Survey Group on request.

## Abbreviations

SOEP: Socio-economic panel survey
SES: Socioeconomic status
SUR: Seemingly unrelated regressions

## References

[1] Adams P, Hurd MD, McFadden D, Merrill A, Ribeiro T (2003). Healthy, wealthy, and wise? Tests for direct causal paths between health and socioeconomic status. J Econ.

[2] Au N, Johnston DW (2014). Self-assessed health: What does it mean and what does it hide?. Soc Sci Med.

[3] Bauldry S, Shanahan MJ, Boardman JD, Miech RA, Macmillan R (2012). A life course model of self-rated health through adolescence and young adulthood. Soc Sci Med.

[4] Brauns H, Scherer S, Steinmann S. The CASMIN educational classification in international comparative research In: Hoffmeyer-Zlotnik J, Wolf C, editors. Advances in cross-national comparison. Kluwer: 2003. p. 221–44.

[5] Ferrer-i-Carbonell A (2005). Income and well-being: an empirical analysis of the comparison income effect. J Publ Econ.

[6] Cervini-Pla M, Castelló J.V (2018). The earnings and employment losses before entering the disability system. Eur J Health Econ.

[7] Chen L, Li W, He J, Wu L, Yan Z, Tang W (2012). Mental health, duration of unemployment, and coping strategy: a cross-sectional study of unemployed migrant workers in eastern China during the economic crisis. BMC Publ Health.

[8] Cleal B, Panton UH, Willaing I, Holt RI (2017). Relative changes in earned income five years after diagnosis with diabetes: A register based study 1996-2012. J Diabetes Complicat.

[9] Cumming G (2014). The new statistics: Why and how. Psychol Sci.

[10] Diener E, Suh E, Lucas R, Smith H (1999). Subjective well-being: Three decades of progress. Psychol Bull.

[11] Dowd JB, Zajacova A (2007). Does the predictive power of self-rated health for subsequent mortality risk vary by socioeconomic status in the US?. Int J Epidemiol.

[12] Easterlin RA (1995). Will raising the incomes of all increase the happiness of all?. J Econ Behav Organ.

[13] Easterlin RA (2001). Income and happiness: Towards a unified theory. Econ J.

[14] Elo IT, Martikainen P, Myrskylä M (2014). Socioeconomic status across the life course and all-cause and cause-specific mortality in Finland. Soc Sci Med.

[15] Fazio RH (2007). Attitudes as object-evaluation associations of varying strength. Soc Cogn.

[16] Fujiwara T, Kawachi I (2008). Social capital and health. Am J Prev Med.

[17] Fujiwara T, Kawachi I (2009). Is education causally related to better health? A, twin fixed-effect study in the USA. Int J Epidemiol.

[18] Gana K, Broc G, Saada Y, Amieva H, Quintard B (2016). Subjective wellbeing and longevity: findings from a 22-year cohort study. J Psychosom Res.

[19] Genolini C, Falissard B (2010). KmL: k-means for longitudinal data. Comput Stat.

[20] Genolini C, Falissard B (2011). Kml: A package to cluster longitudinal data. Comput Methods Prog Biomed.

[21] Gerstorf D, Ram N (2013). Inquiry into terminal decline: Five objectives for future study. Gerontologist.

[22] Gerstorf D, Ram N, Estabrook R, Schupp J, Wagner GG, Lindenberger U (2008). Life satisfaction shows terminal decline in old age: Longitudinal evidence from the German Socio-Economic Panel Study (SOEP). Dev Psychol.

[23] Goebel J, Grabka MM, Liebig S, Kroh M, Richter D, Schröder C, Schupp J (2019). The German Socio-Economic Panel (SOEP). Jahrbücher für Nationalökonomie und Statistik.

[24] Golubev A, Panchenko A, Anisimov V (2018). Applying parametric models to survival data: tradeoffs between statistical significance, biological plausibility, and common sense. Biogerontology.

[25] Henningsen A, Hamann JD. systemfit: A package for estimating systems of simultaneous equations in R. J Stat Softw. 2007;23(4). https://doi.org/10.186372Fjss.v023.i04.

[26] Holt-Lunstad J. Why social relationships are important for physical health: a systems approach to understanding and modifying risk and protection. Ann Rev Psychol. 2017; 69(1). 10.1146/annurev-psych-122216-011902. 10.1146/annurev-psych-122216-011902 29035688

[27] Idler EL, Angel RJ. Self-rated health and mortality in the NHANES epidemiologic follow-up study. Am J Publ Health. 1990; 80(4):446–52. https://doi.org/10.21052Fajph.80.4.446. 10.2105/ajph.80.4.446 PMC1404567 2316767

[28] Idler EL, Benyamini Y (1997). Self-rated health and mortality: A review of twenty-seven community studies. J Health Soc Behav.

[29] Ioannidis JPA (2005). Why most published research findings are false. Plos Med.

[30] Jackson C. flexsurv: A platform for parametric survival modeling in R. J Stat Softw. 2016; 70(8). 10.18637/jss.v070.i08. 10.18637/jss.v070.i08 PMC5868723 29593450

[31] Johnson W, Krueger RF. Genetic effects on physical health: lower at higher income levels. Behav Genet. 2005; 35(5):579–590. 10.1007/s10519-005-3598-0. 10.1007/s10519-005-3598-0 16184486

[32] Jylhä M (2009). What is self-rated health and why does it predict mortality? towards a unified conceptual model. Soc Sci Med.

[33] Kaplan GA, Shema SJ, Leite CMA (2008). Socioeconomic determinants of psychological well-being: the role of income, income change, and income sources during the course of 29 years. Ann Epidemiol.

[34] Krieger N (1994). Epidemiology and the web of causation: Has anyone seen the spider?. Soc Sci Med.

[35] Krieger N, Chen JT, Coull BA, Selby JV (2005). Lifetime socioeconomic position and twins’ health: an analysis of 308 pairs of United States women twins. PLoS Med.

[36] Kroll LE, Lampert T (2009). Socio-economic differences in life expectancy. Data sources in Germany and the potential of the German Socio-Economic Panel Study. Methods Data Anal.

[37] Lawrence EM, Rogers RG, Zajacova A, Wadsworth T (2018). Marital happiness, marital status, health, and longevity. J Happiness Stud.

[38] Lenz K (2006). Sociology of close relationships [Soziologie der Zweierbeziehung] 3 edn.

[39] Lombardo P, Jones W, Wang L, Shen X, Goldner EM. The fundamental association between mental health and life satisfaction: results from successive waves of a canadian national survey. BMC Publ Health. 2018; 18(1). https://doi.org/10.11862Fs12889-018-5235-x. 10.1186/s12889-018-5235-x PMC5848433 29530010

[40] Lorem G, Cook S, Leon DA, Emaus N, Schirmer H. Self-reported health as a predictor of mortality: A cohort study of its relation to other health measurements and observation time. Sci Rep. 2020; 10(1). https://doi.org/10.10382Fs41598-020-61603-0. 10.1038/s41598-020-61603-0 PMC7078209 32184429

[41] Mackenbach JP, Bopp M, Deboosere P, Eikemo TA, Hoffmann R, Kulik MC, Leinsalu M, Martikainen P, Menvielle G, Regidor E, Wojtyniak B, Östergren O, Lundberg O, Kulhánová I (2015). Variations in the relation between education and cause-specific mortality in 19 European populations: A test of the “fundamental causes” theory of social inequalities in health. Soc Sci Med.

[42] Mackenbach JP, Stirbu I, Roskam AJR, Schaap MM, Menvielle G, Leinsalu M, Kunst AE. Socioeconomic inequalities in health in 22 European countries. New Engl J Med. 2008; 358(23):2468–2481. 10.1056/nejmsa0707519. 10.1056/NEJMsa0707519 18525043

[43] McDonough P, Worts D, Corna LM, McMunn A, Sacker A (2017). Later-life employment trajectories and health. Adv Life Course Res.

[44] Michaud PC, van Soest A (2008). Health and wealth of elderly couples: Causality tests using dynamic panel data models. J Health Econ.

[45] Mossakowski KN (2009). The influence of past unemployment duration on symptoms of depression among young women and men in the United States. Am J Publ Health.

[46] OECD: Sickness, disability and work. Paris: OECD Publishing; 2010. 10.1787/9789264088856-en.

[47] Osler M, McGue M, Christensen K (2007). Socioeconomic position and twins’ health: a life-course analysis of 1266 pairs of middle-aged Danish twins. Int J Epidemiol.

[48] Roelfs DJ, Shor E, Davidson KW, Schwartz JE (2011). Losing life and livelihood: A systematic review and meta-analysis of unemployment and all-cause mortality. Soc Sci Med.

[49] Sajjad A, Freak-Poli RL, Hofman A, Roza SJ, Ikram MA, Tiemeier H (2017). Subjective measures of health and all-cause mortality - the Rotterdam Study. Psychol Med.

[50] Senik C (2005). Income distribution and well-being: what can we learn from subjective data?. J Econ Surv.

[51] Singh-Manoux A, Guéguen A, Martikainen P, Ferrie J, Marmot M, Shipley M (2007). Self-rated health and mortality: Short- and long-term associations in the whitehall II study. Psychosomat Med.

[52] SOEP-Group: SOEP-Core v33.1 - PPFAD. SOEP Survey Papers 487: Series D - Variable Description and Coding. Berlin: DIW; 2018. 10.5684/soep.v33.1.

[53] Srivastava V, Dwivedi T. Estimation of seemingly unrelated regression equations. J Econ. 1979; 10(1):15–32. https://doi.org/10.10162F0304-407628792990061-7.

[54] Stowasser T, Heiss F, McFadden D, Wise D, Winter J (2012). “Healthy, wealthy, and wise?” Revisited: an analysis of the causal pathways from socioeconomic status to health. Investigations in the economics of aging.

[55] Tamosiunas A, Sapranaviciute-Zabazlajeva L, Luksiene D, Virviciute D, Peasey A (2019). Psychological well-being and mortality: longitudinal findings from Lithuanian middle-aged and older adults study. Soc Psychiatry Psychiatr Epidemiol.

[56] Torssander J, Almquist YB (2017). Trajectories of economic, work- and health-related disadvantage and subsequent mortality risk: findings from the 1953 Stockholm Birth Cohort. Adv Life Course Res.

[57] Vaillant GE (2003). Mental health. Am J Psychiat.

[58] Vathesatogkit P, Batty GD, Woodward M (2014). Socioeconomic disadvantage and disease-specific mortality in Asia: systematic review with meta-analysis of population-based cohort studies. J Epidemiol Commun Health.

[59] Weber M (1922). Economy and Society [Wirtschaft und Gesellschaft].

[60] Zajacova A, Dowd JB, Schoeni RF, Wallace RB (2015). Employment and income losses among cancer survivors: estimates from a national longitudinal survey of American families. Cancer.

[61] Zellner A (1962). An efficient method of estimating seemingly unrelated regressions and tests for aggregation bias. J Am Stat Assoc.

